# Influence of Aerobic Training and Combinations of Interventions on Cognition and Neuroplasticity after Stroke

**DOI:** 10.3389/fnagi.2016.00164

**Published:** 2016-06-30

**Authors:** Annabelle Constans, Caroline Pin-barre, Jean-Jacques Temprado, Patrick Decherchi, Jérôme Laurin

**Affiliations:** ^1^Aix-Marseille Université, CNRS, ISM, UMR 7287Marseille, France; ^2^Université Nice Sophia Antipolis, LAMHESS, UPRES EA 6309Nice, France

**Keywords:** aging neuroscience, stroke rehabilitation, cerebral ischemia, angiogenesis, cognitive disorders, exercise intensity, neurotrophic factors, rat and human model

## Abstract

Stroke often aggravated age-related cognitive impairments that strongly affect several aspects of quality of life. However, few studies are, to date, focused on rehabilitation strategies that could improve cognition. Among possible interventions, aerobic training is well known to enhance cardiovascular and motor functions but may also induce beneficial effects on cognitive functions. To assess the effectiveness of aerobic training on cognition, it seems necessary to know whether training promotes the neuroplasticity in brain areas involved in cognitive functions. In the present review, we first explore in both human and animal how aerobic training could improve cognition after stroke by highlighting the neuroplasticity mechanisms. Then, we address the potential effect of combinations between aerobic training with other interventions, including resistance exercises and pharmacological treatments. In addition, we postulate that classic recommendations for aerobic training need to be reconsidered to target both cognition and motor recovery because the current guidelines are only focused on cardiovascular and motor recovery. Finally, methodological limitations of training programs and cognitive function assessment are also developed in this review to clarify their effectiveness in stroke patients.

## Introduction

**Figure 1:**
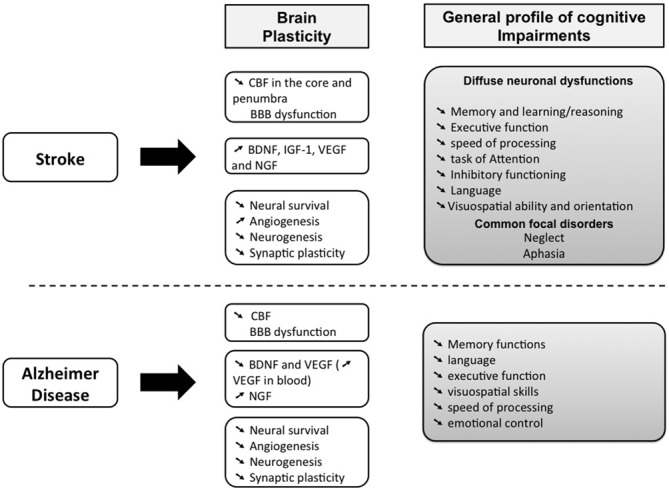
**Comparison of the main cognitive impairments between stroke patient and people with Alzheimer Disease (AD).** Both AD and cerebrovascular disease increase with age. Although the cause of stroke and AD are distinct, we can observe that the cognitive impairments can be confused because these two brain disturbances could lead to dementia. The hypothesis of vascular contributions to cognitive impairment and dementia (VCID) is that cognitive impairments result from cerebrovascular damages and thus cover the effect of AD, cerebrovascular diseases, including stroke, on cognition (Corriveau et al., 2016). It can be added that more stroke is severe, more the risk of dementia is high. Micro-infarct, silent stroke or small vessel insults are often related to mild cognitive impairments (see also McDonnell et al., 2011; Cumming et al., 2012). However, the outcomes on brain plasticity is specific to each brain disorders (Sopova et al., 2014).

Sedentary older adults are prone to cardiovascular diseases, such as stroke (Bherer et al., 2013), which occurs when blood flow is interrupted to a part of the brain. This trauma leads to severe motor dysfunctions and it may also aggravate cognitive impairments resulting from normal aging (Rafnsson et al., 2007, 2009; Deary et al., 2009; Waldstein and Wendell, 2010). Indeed, stroke survivors have more than twice the risk of subsequently developing dementia compared with people who have never had a stroke (Tatemichi et al., 1992; Patel et al., 2002). For instance, a stroke situated on the left hemisphere might disturb language and comprehension, which reduce the ability to communicate (Karbe et al., 1990; Pirmoradi et al., 2016). When the right hemisphere is affected, the intuitive thinking, reasoning, solving problems as well as the perception, judgment and the visual-spatial functions could be impaired (Tatemichi et al., 1994; Patel et al., 2002; Cumming et al., 2012; Sun et al., 2014b; Harris et al., 2015; Tiozzo et al., 2015; Save-Pédebos et al., 2016). It makes thus difficult for patients to locate objects, walk up or down stairs or get dressed. Consequently, cognitive disorders are one of the strongest predictor of the inability to return to work, that contribute to the socioeconomic burden of stroke (Kauranen et al., 2013). However, stroke-induced cognitive disorders are often underestimated relative to motor impairments because they are confused with pre-existing symptoms of age-related mild cognitive impairments or Alzheimer’s Disease (AD; Figure 1; Sun et al., 2014b; Corriveau et al., 2016). Furthermore, cognitive impairments are frequently associated with poor motor recovery (Patel et al., 2002; Leśniak et al., 2008; Rand et al., 2010). It suggests that stroke-induced cognitive dysfunctions and brain plasticity might also affect the stability, flexibility and learning of complex movements (e.g., locomotion, unimanual aiming, bimanual coordination), in which cognitive resources are highly involved as it was already observed in older adults (Temprado et al., 1999, 2013; Sleimen-Malkoun et al., 2012, 2013; Cohen et al., 2016).

It is thus of great importance to find effective interventions to induce both motor and cognitive improvement after stroke. In this respect, it is now widely established that aerobic training enhances cardiorespiratory fitness, muscular endurance and functional recovery of stroke patients resulting in a higher quality of life (Ivey et al., 2005; Macko et al., 2005; Marsden et al., 2013). Over the past few years, few studies have also shown that aerobic training could improve cognitive functions and promotes neuroplasticity in stroke patients (Quaney et al., 2009; Rand et al., 2010; Wogensen et al., 2015). In support of these observations, animal studies have revealed that such training effects on cognitive functions might be partially mediated by the release of neurotrophic factors that promotes angiogenesis, neurogenesis, synaptogenesis and synthesis of neurotransmitters that could not be directly investigated at central level in humans (Churchill et al., 2002; Cotman et al., 2007; Lövdén et al., 2010). However, despite firm evidence supporting the use of aerobic exercise for stroke patients, the mechanisms underlying neuroplasticity that is at origin of cognitive and motor recovery in stroke patients remain unknown.

In the present review, we first examine how aerobic training might play a beneficial role on cognition after stroke. In particular, we highlight the influence of aerobic training on neuroplasticity in both human and animal. We also discuss whether additional rehabilitation strategies and/or pharmacological treatments could accentuate neuroplasticity and consequently, the recovery of cognitive functions. In addition, we present the different exercise parameters that should be taken into account in aerobic training, namely: intensity, duration, frequency (number of session per week) and types of exercise as well as timing of training initiation after stroke onset. Indeed, they might differ in their effectiveness to improve cognition and motor functions. Therefore, classic recommendations for aerobic exercise are reconsidered to target cognition as well as motor and cardiovascular functions. Finally, we discuss about the methodological limitations that may hamper the understanding of aerobic training effects on cognition both in human and animal studies.

## Influence of Aerobic Training on Neuroplasticity and Cognitive Functions

Cognitive disorders (i.e., executive function and/or information processing speed), motor dysfunctions (i.e., locomotion, balance and strength impairments) and cardiorespiratory fitness weakness are frequently observed in both elderly and stroke people. However, the severity of these impairments is exacerbated due to ischemia in older stroke patients (Gordon et al., 2008; Billinger et al., 2012; Cumming et al., 2013; Harada et al., 2013). Moreover, some neural mechanisms involved in cognitive disorders appear to be close between aging and cerebral ischemia. Specifically, a decrease of neurotrophic factor release (Ang et al., 2003; Silhol et al., 2005; Chae and Kim, 2009) or an abnormal level of oxidative stress and inflammation in hippocampus have been observed in both population (Joseph et al., 2005; Wang et al., 2007). In addition, cerebral blood flow decreases in ischemic region (Cupini et al., 2001; Zhang et al., 2013a) while the reduced vessel density in aging brain leads to an overall reduced blood flow and oxygenation into the brain (Petcu et al., 2010). Therefore, promoting angiogenesis might be critical for these two populations.

In numerous studies, it was demonstrated that aerobic training could induce beneficial effects on brain plasticity and associated cognitive functions as well as motor and cardiorespiratory functions in aging population (Patel et al., 2002; McAuley et al., 2004; Kramer and Erickson, 2007; Leśniak et al., 2008; Erickson and Kramer, 2009). Thus, it can be hypothesized that such training could lead to similar positive effects on cognitive functions in stroke patients. In the following, we present evidence supporting this hypothesis in both older adults and animal studies. Then, on the basis of the few available studies in the literature, we made an overview of the effects of aerobic training on cognitive functions in people and animal with cerebral ischemia.

### Studies on Healthy Older Adults

It is now recognized that aerobic training stimulates the positive plasticity of the aging brain. Such exercise-related plasticity mediates the maintenance, or even the increase, of cognitive performance as indicated by the improvement of executive functions and long-term memory. Such enhancements were observed in spite of the heterogeneity of methods, subject characteristics, training parameters or cognitive tasks used in the related studies (Colcombe and Kramer, 2003). Thanks to the use of sophisticated brain imaging technologies, cerebral modifications induced by aerobic training has been observed at both structural (i.e., increase in white and gray matter volumes and changes in synaptic connections) and functional (i.e., changes in brain activation patterns) levels (Churchill et al., 2002; Cotman and Berchtold, 2002; Colcombe and Kramer, 2003; Colcombe et al., 2004a,b, 2006; McAuley et al., 2004; Erickson and Kramer, 2009; Greenwood and Parasuraman, 2010; Voss et al., 2010a,b). For instance, using functional magnetic resonance imagery (fMRI), it was shown that elderly people aerobically trained displayed a higher activation of brain areas involved in attentional control and inhibitory functioning, while a reduced activation is observed in brain areas involved in behavioral conflict (Colcombe et al., 2004a). Additionally, 12-week of both bike and treadmill aerobic training increases the hippocampal and anterior cingulate cortex cerebral blood flow and also an immediate and delayed verbal memory. Such increase is closely related to neuroregeneration (Petcu et al., 2010; Chapman et al., 2013; Duzel et al., 2016). This result was recently reinforced by showing with the use of gadolinium-based perfusion imaging (3 Tesla MRI) that aerobic fitness improvement in healthy older adults is correlated with changes in hippocampal perfusion and volume that were positively related to changes in recognition memory and early recall for complex spatial objects (Maass et al., 2015).

Circulating neurotrophic factor measurements such as brain-derived neurotrophic factor (BDNF), insulin-like growth factor-1 (IGF-1) and vascular endothelial growth factor (VEGF; see below for details) might also explain the influence of aerobic training on neuroplasticity and cognition in Human but this point remains controversial (Cotman et al., 2007). In the one hand, few studies have observed an increase in serum BDNF level in older adults after 1-year of aerobic training that was correlated with an increase in hippocampal volume and improvement in spatial memory performance as well as in executive function (Erickson et al., 2012; Leckie et al., 2014). Specifically, Leckie et al. (2014), revealed that the oldest subjects exhibited the greatest increase in circulating BDNF levels and improvement in task performance after training. This observation suggests that age affects BDNF serum levels (Leckie et al., 2014). On the other hand, numerous studies did not provide robust evidence for enhancement of these neurotrophic factors after several weeks of training in aging people (Voss et al., 2013; Maass et al., 2015). For instance, serum BDNF, VEGF and IGF-1 level did not increase after 12-month of aerobic or non-aerobic (stretching exercises) training despite an increase of connectivity in the temporal lobe between the bilateral parahippocampus and the bilateral middle temporal gyrus (Voss et al., 2013). Recently, Maass et al. (2015), confirmed these results by showing that no difference in changes of circulating BDNF, VEGF and IGF-1 was observed after 3-month of treadmill (training group) or progressive-muscle relaxation/stretching (control group) despite an increase of hippocampal perfusion and volume (Maass et al., 2015). On the basis of these results, it was suggested that the training-related improvement of cerebral perfusion could lead to beneficial effects on neural function without any contribution of growth factors.

These contradictory findings might result from limitations associated with the circulating serum growth factor measurements and training protocols. For instance, nutritional status and changes in energy balance inducing by exercise interventions might affect growth factor levels (Monteleone et al., 2004). Moreover, as it was previously mentioned (Coelho et al., 2013), studies presented different training durations and exercise intensity and heterogeneous samples. Authors also included a different proportion of females and/or males in their studies. Such imbalance in gender composition of experimental groups could have an effect of baseline serum BDNF that might hide possible influence of aerobic training (Komulainen et al., 2008; Driscoll et al., 2012). It has also been suggested that the variable time-windows of circulating growth factor measurements and the analysis techniques used for quantifying blood growth factor levels could also affect the results and need to be clarified (Maass et al., 2015). Finally, data from circulating growth factor assessment should be interpreted with caution since no direct relationships between circulating and brain growth factor levels has been found in human. Studies on human only demonstrated that peripheral BDNF was associated with cognitive performance and cerebral structure integrity (Ventriglia et al., 2013). However, it was also established in animal model that BDNF, VEGF and IGF-1 might have the potential to cross the blood-brain barrier (BBB) in both directions in the central nervous system (CNS; Pan et al., 1998; Cotman et al., 2007). As an illustration, Karege et al. (2002), demonstrated that serum BDNF was positively correlated with cortical BDNF in rat model. In addition, blocking the peripheral IGF-1 or VEGF access to the brain impeded exercise-induced plasticity in the hippocampus (Karege et al., 2002; Cotman et al., 2007). However, other studies have shown in neurological disorders such as stroke and depression that changes in regional brain BDNF levels are not associated with changes of BDNF levels in peripheral blood (Elfving et al., 2010; Béjot et al., 2011). To date, it thus remains difficult to clearly determine the real role of circulating growth factors on cognitive function in older subjects.

### Studies on Healthy Animals

Animal experiments could provide information about neuroplasticity mechanisms at cellular level in brain areas involved in cognitive functions. To date, three main neurotrophic factors have been identified to contribute to increase neuroplasticity after aerobic training: (i) the VEGF, which is a protein whose main role is to stimulate angiogenesis; (ii) the BDNF, which is a critical protein involved in brain plasticity. Indeed, this neurotrophin mediates numerous proteins expressions and molecular pathways, i.e., synapsin-I and synpatophysin, both involved in synaptic transmission and neurotransmitter release, C-AMP response element-binding protein (CREB) and beta calcium/calmodulinedependent kinase II (β-CaMKII) both contributing in long-term potentiation (LTP). BDNF thus promotes synaptic plasticity, neurogenesis and neuronal survival within the hippocampus (Vaynman et al., 2004; Cassilhas et al., 2012); and (iii) the IGF-1, which is a peptide that crosses the BBB and stimulates neurogenesis and angiogenesis. These growth factors act together to promote training-related benefits in brain plasticity and associated cognitive functions.

It was found in adult rats that aerobic training induced-angiogenesis is associated with an increase in brain VEGF mRNA and protein (Ding et al., 2006; Cotman et al., 2007). Moreover, Swain et al. (2003), have proved that prolonged voluntary exercise induced an increase of blood volume in the motor cortex of 19% greater than control animals (Swain et al., 2003). Angiogenesis needs to be promoted because some animal studies have suggested that angiogenesis was closely linked to adult neurogenesis and other neuroplasticity mechanisms (Pereira et al., 2007).

It was demonstrated that 8-week of aerobic training on treadmill induced an improvement in learning speed and spatial learning. Authors suggested that these results could be explained by an increase of hippocampal BDNF and IGF-1 and more precisely by the activation of BDNF/TrkB/β-CaMKII pathway and to a lesser extent to IGF-1/IGF-1R/Akt (also known as protein kinase B) pathway (Cassilhas et al., 2012). Radák et al. (2001, 2006) indicated that short- and long-term memory was improved after 8 weeks of regular swimming exercise in middle-aged rats (14-month-old). BDNF and nerve growth factor (NGF) expressions were up-regulated as well as a reduction of accumulation of reactive carbonyl derivatives that are known to damage proteins, nucleic acids ad lipids (Radák et al., 2001, 2006). Similarly, one study on aging rat model, induced by D-galactose injection, has shown that 8-week of moderate aerobic training treadmill could importantly increase both level of NGF and his receptor, the tyrosine kinase A (TrkA) in the hippocampus. NGF/TrkA actives the phosphoinositide 3-kinase (PI3-K)/Akt pathway that decreases apoptosis (Wiesmann and de Vos, 2001; Chae and Kim, 2009). Excessive reactive oxygen species (ROS) production can also trigger apoptosis in brain areas such as the hippocampus that contribute to neurodegenerative disorders and cognitive function impairments in aging people. However, moderate intensity training on treadmill (18 m/min, 30 min/day during 15 weeks) in middle-age female rats (12-month-old) could enhance antioxidant defense system and thus induced a neuroprotective effect in the hippocampus (Marosi et al., 2012). Training also facilitated the release of metabolic proteins in the hippocampus such as mitochondrial precursor of ornithine aminotransferase and isocitrate deshydrogenase subunit alpha. However, these results remain difficult to interpret because authors suggested that alteration of mitochondrial proteins may be either reflecting mitochondrial damage or adaptation to mitochondrial dysfunction (Kirchner et al., 2008).

### Studies on Stroke Patients

Only three studies investigated the role of aerobic training on cognition in stroke patients (Table 1). Two of them indicated that aerobic training might reduce cognitive disorders by improving functional outcomes as well as motor learning by the increase of information processing speed and implicit memory while executive functions remain affected (Quaney et al., 2009; El-Tamawy et al., 2014). However, patients did not preserve long-term beneficial effects 8 weeks after the end of aerobic training (Quaney et al., 2009). Contrary to what was observed in healthy subject, an acute and short aerobic exercise on treadmill (20 min; 70% of the heart rate reserve) did not induce cognitive improvement in stroke patient while a slightly improvement of the movement of upper-extremity was observed (Ploughman et al., 2008).

**Table 1:** **Summary of aerobic training protocols with or without combination (pharmacological agents or other types of exercise) and their effects on cognition in stroke patient**.

Studies	Participants	Aerobic training	Combination	Cognitive assessments	Results
Ploughman et al. (2008)	*n* = 21; (61.4 ± 10.2 years old); 6 months to 5 years post-stroke.	- AEX: single bout of 20 min on BWSTT at 70% of THR or 13 on The Borg RPE Scale. - Control condition: 20 min review of home exercises.	none	- TMT-A and B - Symbol digit substitution test - Paced auditory serial addition test	No significant improvement observed No relationship between exercise intensity and cognitive tests.
Quaney et al. (2009)	*n* = 38 (58.96 ± 14.68 and 64.10 ± 12.30 years old);> 6 months post-stroke.	- AEX: 45 min on cyclo-ergometer 3 times/week for 8 weeks at 70% HRmax (*n* = 19). - Control group: Upper- and lower-extremities stretching activities 45 min on cyclo-ergometer 3 times/week for 8 weeks at home (*n* = 19).	none	- WCST - Stroop task - TMT-A and B - SRTT test - PGFM	**↗** VO_2peak_ **↗** Complex motor learning tasksNo significant improvement observed of executive function measures
Rand et al. (2010)	*n* = 11 (67 ± 10.8 years old); > 4 years post-stroke.	AEX: 1 h composed of stretching, balance, task specifics exercises and 20 min of moderate exercise on The Borg RPE Scale (somewhat hard), 2 times/week for 6 months.	1 h of recreation time, 2 times/week for 6 months.	- Digit backwards - TMT - RAVLT - WWT - Digit symbol - Stroop test	**↗** Dual task, response inhibition and memory at 3 months **↗** Executive functions **↗** Response inhibition at 6 months **↗** VO_2_ max at 3 and 6 months
Kluding et al. (2011)	*n* = 9 (63.7 ± 9.1 years old); > 6 months post-stroke.	AEX: 1 h on TBRS,3 times/week for 12 weeks.	Lower extremity muscle strengthening exercises: 1 h, 3 times/week for 12 weeks.	- Digit span backwards task - Flancker test - Memory component of the SIS	**↗** Digit span backwards task **↗** SIS total score (including memory subscale) Significant relationship between improvement of aerobic and Flancker tests
Marzolini et al. (2013)	*n* = 41 (63.6 ± 13.5 years old); 10 weeks post-stroke (74 ± 135 days).	AEX: treadmill or cyclo-ergometer depending on patient ability, 20–60 min, 5 times/week for 6 months at 40–70% of HRR or VO_2peak_, HR achieved at the ATge and/or 11–6 on the RPE of the Borg Scale. Intensity was progressively increased.	Resistance training during 6 months: - 50% to 60% of 1RM for non-affected limb; - ≥ 50% of 1RM and/or 13–14 RPE on the last repetition on the affected limb. - Intensity from 10–15 repetitions.	MoCA (7 different cognitive domains): - Visuo-spatial and executive function - Attention and concentration - Language - Delayed recall - Orientation - Abstraction - Naming	**↗** VO_2peak_ **↗** VO_2_ at the ATge **↗** Upper- and lower-muscular strength **↗** Attention and concentration **↗** Visuo-spatial and executive function
El-Tamawy et al. (2014)	*n* = 30 (49.67 ± 6.98 and 48.4 ± 6.39 years old); 3–18 months post-stroke.	AEX: 30 min, 3 times/week for 8 weeks of physiotherapy program + 45 min cyclo-ergometer Control group: 30 min, 3 times/week for 8 weeks of physiotherapy program	none	- ACER test	**↗** serum BDNF level **↗** ACER for AEX groupSignificant correlation between changes in serum BDNF level and in ACER test
Bartolo et al. (2015)	*n* = 47 (64.5–67 years old); Within the previous 2 weeks post-stroke.	L-deprenyl (*n* = 23) Placebo group (*n* = 24) Both group: 1 h of physiotherapy (6 days/week) + 30 min of cyclo/arm-ergometer training (5 days/week) + 1 h of occupational therapy (3 days/week) during 6 weeks.	L-deprenyl: 10 mg/day once day during 6 weeks.	- TMT-A and B - MoCA and RAVLT - Logical memory - Digit Span - Corsi’s test - Attentive matrices - Stroop test - Symbol digit - PM-47 and FAB - Phonological fluency - Semantic fluency	**↗** Logical memory **↗** Attentive matrices **↗** TMT-A and B **↗** PM-47 **↗** MoCA **↗** Digit Span **↗** Stroop test

### Studies on Animal with Cerebral Ischemia

**Figure 2:**
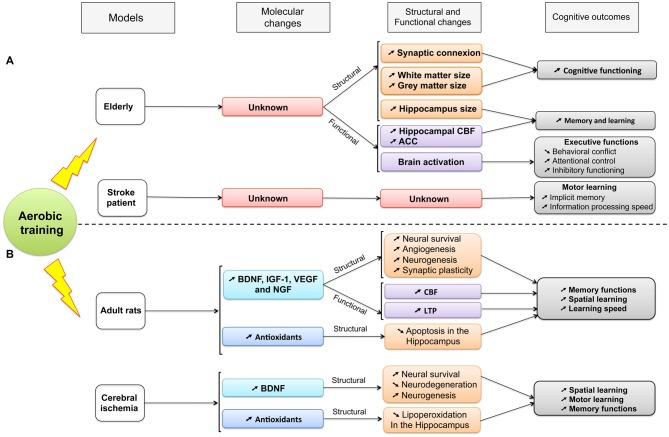
**A summary of the established effects of aerobic training on molecular, structural and functional changes and their consequences on cognitive functions (see details in the text).** Different models are compared: aging and stroke in both **(A)** human and **(B)** animal populations. ACC, Anterior Cingulate Cortex; BDNF, Brain-Derived Neurotrophic Factor; CBF, Cerebral Blood Flow; IGF-1, Insulin Growth Factor-1; LTP, Long-term potentiation; NGF, Nerve Growth Factor; VEGF, Vascular Endothelial Growth Factor.

Consistent with healthy older animals, neuroplasticity can be partially promoted by aerobic training through up-regulation of the expression of BDNF, synapsin-I and post-synpatic density protein 95 (PSD-95), also involved in LTP within hippocampus (dentate gyrus, CA1 and CA3 areas) and by increase of both CREB phosphorylation and newborn cell survival (Luo et al., 2007; Shih et al., 2013). The beneficial effect of training was reinforced when the activation of the high affinity BDNF receptor, the TrkB, was blocked because the function of BDNF was reduced and the exercise-induced spatial learning enhancement was impeded (Griesbach et al., 2009). Thus, consistent with data collected within aging rodents, it is supposed that BDNF-mediated pathway contributed to explain the improvement of spatial memory performances after cerebral ischemia. Furthermore, spatial memory performances were positively correlated with an increase of both newborn cell survival in bilateral dentate gyrus and restored microtubule-associated protein 2 (MAP2) density after cerebral ischemia (Luo et al., 2007; Shimada et al., 2013). Treadmill training might also protect against cognitive impairments in rats with bilateral common carotid artery occlusion (CCAO) by reducing the lipoperoxidation in the hippocampus through an increase of antioxidant capacity and by improving the acquisition of a spatial task as well as the performance for both retention and working memory (Figure 2; Cechetti et al., 2012). In addition, few weeks of treadmill training in rat with cerebral ischemia increases the VEGF, and its regulatory protein, caveolin-1, and improves the cerebral blood flow in ischemic region (Zhang et al., 2013a; Gao et al., 2014). However, no study has directly shown that vascular changes could contribute to explaining the cognitive disorders.

## Is the Combination of Aerobic Training with Other Types of Therapies Effective for Improving Cognition Recovery After Stroke?

### Combination with Other Rehabilitation Exercises

The question arises of whether different types of exercise could be combined to improve the effects that they could have separately. As reviewed by Chang et al. (2012), resistance exercise alone could positively impact selective cognitive functions in older adult such as information-processing speed, attention and several aspects of memory and executive functions (Chang et al., 2012). It was postulated that the main potential underlying mechanism for these benefits is based on resistance training-induced IGF-1 both in brain and blood circulation. Moreover, growing evidence have shown that the combination of aerobic and resistance training induced superior beneficial effect on cognition than aerobic exercise alone in aging population (Colcombe and Kramer, 2003). In this way, the association between resistance and aerobic training might be a potential strategy to further facilitate the cognitive function recovery in stroke population. However, few studies have been conducted to test this attractive hypothesis (Rand et al., 2010; Kluding et al., 2011; Marzolini et al., 2013). Results indicated that combination of aerobic training and lower extremity muscle strengthening improved executive functions, attention and voluntary motor control (Kluding et al., 2011). This is consistent with the study carried out by Marzolini et al. (2013), in which 6 months of both aerobic training at moderate intensity associated with resistance exercises induced improvements in the Montreal Cognitive Assessment (MoCA) scores in subdomain of attention/concentration and visuospatial/executive functioning (Marzolini et al., 2013). Another study on chronic stroke patients combining aerobic training with stretching, balance and task-specific exercises as well as sessions of recreation time has shown an improvement in verbal memory and cognitive flexibility but did not promote executive function (Rand et al., 2010). However, since the isolated effects of resistance and aerobic training were not compared with the combination of both of them, it remains difficult to ensure that such combination induces superior beneficial effects on cognition than aerobic intervention alone after stroke. It should also be noticed that the neuroplasticity associated with cognition recovery after combination of aerobic and other rehabilitation strategies has not been investigated in both human and animal stroke studies.

### Combination of Aerobic Training with Pharmacological Treatments

Currently, both aerobic and behavioral training have been shown to improve cognition on stroke patient but it remains insufficient to induce total recovery (Quaney et al., 2009; Cherry et al., 2014). Thus, it seems crucial to find new strategy combinations including aerobic exercise to amplify the effects on cognition. Many pharmacological treatments are possible candidates to improve cognitive function after stroke by stimulating brain plasticity. For instance, the drug memantine, used for the treatment of moderate to severe dementia of the Alzheimer’s type, could increase synaptic plasticity and LTP and decrease overactivation of N-methyl-D-aspartate-type glutamate receptors (NMDA-r) in order to limit excitotoxic neuronal death (Martin and Wang, 2010; Berthier et al., 2011). Other substances, like citicoline, an intermediate in the generation of phosphatidylcholine from choline, could increase the level of acetylcholine and dopamine as well as decrease infarct volume in animal model with cerebral ischemia (Alvarez-Sabín and Román, 2011).

To date, only the intravenous (IV) recombinant tissue plasminogen activator (rt-PA) was approval by the USA Food and Drug Administration for the treatment of acute ischemic stroke in human (Marshall, 2015). The rt-PA is focused on thrombolytic events and could reduce the infarct volume (Nys et al., 2006) by restoring cerebral blood flow and oxygen supply to ischemic brain tissue (Thompson and Ronaldson, 2014). When rt-PA is administrated within the first 3 h after stroke, it could improve clinical outcomes and activities of daily life from 3, 6 and 12 months (Kwiatkowski et al., 1999; Nys et al., 2006; Murao et al., 2014). However, the effects of rt-PA administration during the first 24 h on cognitive functions are limited to aphasia that is explained by the reperfusion of language areas (only for one-third of patient; Nys et al., 2006; Hajjar et al., 2013; Kremer et al., 2013). Only one study used rt-PA in combination with physical activity during leisure-time. Results showed that combination of the 2 strategies did not accentuate the cognitive and physical improvements compared to rt-PA alone after 3 months (Decourcelle et al., 2015).

In preclinical study, L-deprenyl is a pharmacological treatment used to decrease ROS known to contribute to neuronal cell death in the ischemic core following permanent middle cerebral artery occlusion in mice (Unal et al., 2001). This substance has already shown the clinical effectiveness on cognitive functions in the elderly human with AD (Wilcock et al., 2002). L-deprenyl generates an amphetamine-like effect by acting on the release of dopamine and hampering its reuptake that contribute to the modulation of both attention and executive functions (Table 1; Bartolo et al., 2015). After stroke, combination of both L-deprenyl and standard rehabilitation including physiotherapy, cycle arm-ergometer training and occupational therapy during 6 weeks showed an improvement of logical memory, visual selective attention and non-verbal reasoning (Bartolo et al., 2015). However, this study did not compare the effect of such combination with the L-deprenyl treatment effect or the standard rehabilitation alone. Therefore, it remains difficult to postulate that the combination of these two strategies reinforced the cognitive recovery. In rat model, D-amphetamine administration combined to locomotor training enhances locomotor recovery after cortical injury (Feeney et al., 1982; Ramic et al., 2006). Moreover, other study assessed the effect of D-amphetamine administration combined with functional rehabilitation and physical therapy alone on cognitive memory performance and motor recovery after embolic stroke. Results showed that D-amphetamine with functional rehabilitation was more effective to improve cognitive performance than training alone, especially for memory, but the latter could improve fine motor performance (Rasmussen et al., 2006). By contrast, it was found in stroke patient that the combination of unique training session of—lower-extremity stability platform task, upper-extremity simulated feeding task and declarative learning—with D-cycloserine treatment did not accentuate the effect of motor and cognitive training (Cherry et al., 2014). This agent is already known to improve both motor and cognitive functions through increasing LTP in the hippocampus by regulating NMDA-r (Yaka et al., 2007). Cherry et al. (2014) postulated that an unique training session is not enough to amplify pharmacological treatment and that D-cycloserine could not act on the reduced NMDA-r function, and thus, on the motor task learning after stroke.

Finally, the review of literature suggests that no efficient combination between pharmacological treatments and training has yet found to improve recovery on cognitive functions. However, several combinations of pharmacological treatments and aerobic training have proven effectiveness on motor recovery after cerebral ischemia (Pin-Barre and Laurin, 2015). For example, the combination of both S-nitrosoglutathione and training accentuated neuroplasticity (reduce excitotoxicity, inflammation and protect BBB integrity) and motor recovery (Sakakima et al., 2012). Future studies should investigate the effects of these types of associations on cognitive recovery.

## Motor Vs. Cognitive Function Recovery: Can Aerobic Training Similarly Facilitate Both?

Currently, the recommendations for the use of aerobic training after stroke were only established according to the effectiveness of endurance programs on cardiovascular and motor functions (Pang et al., 2013; Billinger et al., 2014). The cognitive outcomes are thus not taken into account for prescribing physical exercise. However, several findings arising from rodent studies indicate that the most appropriate intensity, the optimal timing to initiate training and the exercise mode (type of exercise) for improving motor performance might be different to improve cognitive function and motor recovery.

### Cognitive Performance Improvement by Aerobic Training

#### Exercise Intensity

Low-intensity aerobic training seems to be more effective than high-intensity training to promote cognitive health. Indeed, animal studies have shown that low-intensity training induced superior positive effects on spatial learning task in a water maze test and on both object recognition and location tasks than intense training (Shih et al., 2013; Shimada et al., 2013). Such cognitive task improvements were concomitant with an increase in plasticity-related proteins such as the increase of hippocampal BDNF level, synapsin-I as well as the number of dendritic spines and the number of neurons in the ipsilateral dentate gyrus (Shen et al., 2013; Shih et al., 2013; Shimada et al., 2013). It was mainly explained by the fact that, compared to low-intensity exercise, the high-intensity exercise induced higher levels of stress-hormone, which might downregulate BDNF level in hippocampus (Schaaf et al., 1999; Soya et al., 2007). Nevertheless, caution is required concerning the chosen running intensity. Indeed, the speed, fixed to 20–22 m/min, was considered as “high” intensity in Shih and Shimada’s studies (Shih et al., 2013; Shimada et al., 2013) while others have classified such range of speed as moderate intensity (Sun et al., 2008; Ni et al., 2012). Therefore, the real influence of exercise intensity based on physiological parameters (for example, lactate threshold or VO_2peak_) on cognitive recovery remains to be further investigated (see “Methodological Considerations” Section).

#### Timing of Training Initiation

Late exercise, beginning after the first week post-brain injury, is more effective than early exercise (starting during the first week) to improve cognitive functions. Indeed, spatial learning and retention were better improved with the late training and was associated with the upregulation of BDNF (Griesbach et al., 2004; Clark et al., 2008). Voluntary running wheel training, starting 1 week after transient focal cerebral ischemia in mice, promoted neurogenesis in the adult dentate gyrus and spatial memory rebuilding (Luo et al., 2007). Interestingly, the Schmidt meta-analysis indicated that a start of skilled forelimb training from days 1 to 5 post-injury was more effective to improve cognitive function compared to late training (Bland et al., 2000; Wurm et al., 2007; Schmidt et al., 2014). It might be suggested that motor skilled training might start before aerobic training to improve cognitive functions.

#### Forced vs. Voluntary Training

The training mode might also influence the neuroplasticity underlying cognitive performance. Some studies have recently demonstrated that involuntary exercise (functional electrical stimulation), forced (treadmill) and voluntary (running wheel) training have similar beneficial effects on cognitive function after cerebral ischemia as indicated by improvement of both object recognition and location tests (Lin et al., 2015). Moreover, these three training regimens enhanced the levels of synapsin I, synaptophysin, PSD-95, MAP-2 and Tau protein in the hippocampus. It was confirmed by a previous study on healthy rats where 6 weeks of forced swimming or voluntary running resulted in similar increase of hippocampal BDNF level and in similar effect on learning capabilities and short/long term memories (Alomari et al., 2013). Nevertheless, other studies indicated divergent results. On one side, Hayes et al. (2008), demonstrated that, after 2-h of middle cerebral artery occlusion, forced treadmill training reduced infarct volume and increased the gene expression of heat shock proteins (Hsp), in particular the 27 kDa Hsp and the 70 kDa Hsp mRNA than voluntary exercise despite higher corticosterone level. The Hsp acts in the brain as molecular chaper ones with neuroprotective activities (Hayes et al., 2008). Other authors indicated that 12 weeks of forced treadmill training could protect against cognitive and biochemical impairments caused by CCAO in rat (Cechetti et al., 2012). Similar results were found after whole-brain irradiation where forced running training reduced the neurocognitive deficits but also the hippocampal neurogenesis impairments, i.e., the down-regulation of BDNF-mediated pathway (including TrkB receptors, Akt and CREB, for example; Ji et al., 2014). On other side, some authors indicated that voluntary exercise is the most effective training in up-regulating the hippocampal BDNF level (Luo et al., 2007; Ke et al., 2011). Indeed, Ke et al. (2011), compared the effect of voluntary, involuntary and forced training after cerebral ischemia in rat. Results indicated that 7-day intervention of voluntary training induced higher level of BDNF in the hippocampus than the other modes (Ke et al., 2011). Such divergent results might be attributable to variable experimental designs.

### Motor Performance Improvement by Aerobic Training

#### Exercise Intensity

To date, post-stroke guidelines recommend moderate-intensity continuous aerobic training to improve aerobic capacity and motor recovery (40–80% of the maximum heart rate reserve; 3–5 sessions/week; 20–60 min/session). However, it was demonstrated that high-intensity exercise could improve aerobic fitness by increasing the peak oxygen uptake (VO_2peak_) and 6-min walk performances, that remained higher 1 year after the end of training compared with baseline value (Globas et al., 2012). In addition, it has been found that the high-intensity interval training (HIT) could promote superior beneficial cardiovascular and muscular adaptations among persons with different cardiorespiratory disorders (Rognmo et al., 2004; Wisløff et al., 2007). HIT is defined as repeated series of brief and intense exercise separated by active or passive rest periods. This type of intense training is also well known to be a time-efficient strategy to promote metabolic adaptations because the total session duration is strongly reduced compared to traditional moderate-intensity continuous aerobic training (Sun et al., 2014b). Such finding is important given that “lack of time” remains the most cited barrier to regular aerobic exercise participation.

It was observed that HIT is well accepted for ambulatory chronic stroke and could induce encouraging improvements of quality of life as observed by improvements of VO_2peak_ and work economy (Gjellesvik et al., 2012; Boyne et al., 2013; Mattlage et al., 2013). Recently, only one study has compared HIT (series of 30 s at maximum tolerated treadmill speed separated by 30–60 s rest periods) and traditional moderate-intensity continuous aerobic training (Boyne et al., 2016). Authors indicated that no clear difference between HIT and moderate-intensity continuous aerobic training was observed because of a small sample size. Nevertheless, this type of training seems to be feasible and safe because no adverse events occurred.

#### Timing of Training Initiation

It was postulated in human studies that starting rehabilitation program in the acute-subacute phase after stroke could prevent complications relating to prolonged inactivity (i.e., deconditioning period) and presented a low relative risk for adverse effects (Musicco et al., 2003). Numerous studies in rodents with cerebral ischemia have also observed that an early starting aerobic exercise (from days 1 to 5) was more effective to improve the running performance and to reduce the infarct volume compared to late training (Bland et al., 2000; Luo et al., 2007; Schmidt et al., 2014). For example, Park et al. (2010), indicated that early treadmill training could better improve the motor performance, using the Rotarod test, than late treadmill training after hemorrhagic stroke (Park et al., 2010). Such early training did not increase the infarct volume or brain edema in accordance to other studies (Matsuda et al., 2011; Zhang et al., 2013b). Likewise, an early treadmill exercise increased the cellular expression levels of some neurotrophic factors, promoted cell growth and reduced the expression of apoptosis markers (Mizutani et al., 2010; Matsuda et al., 2011). Moreover, an early endurance exercise improved blood flow in the ischemic region and promoted angiogenesis (Zhang et al., 2013b). We may also add that sensorimotor deficits and cortical infarct volume were aggravated on a longer-term when training started too soon i.e., before 24 h post-ischemia (Kozlowski et al., 1996; Risedal et al., 1999; Bland et al., 2000; DeBow et al., 2004; Schmidt et al., 2014).

#### Forced vs. Voluntary Training

Numerous studies highlighted that forced treadmill training is more effective than all the other types of exercise, included voluntary exercise, to improve running function, aerobic fitness and to reduce infarct volume (Takamatsu et al., 2010, 2016; Schmidt et al., 2014). However, some authors indicated that voluntary exercise is more effective to improve motor recovery using the De Ryck’s behavioral test (Ke et al., 2011). These controversial results might be explained by the use of different motor behavioral tests between studies as well as by a different training protocol (variable speed and timing of training initiation).

#### Concomitant Improvement of Cognitive and Motor Functions Induced by Aerobic Exercise

Interestingly, Sun et al. (2014), might find a compromise in rats by proving that training with gradually increased intensity on treadmill (from 5 to 26 m/min) could better improve motor function and produce higher hippocampal BDNF with lower stress compared to both stably low and high intensity training (Sun et al., 2014a). These results were in accordance with Zhang et al. (2012), study in which both motor performance (forelimb placing, stepping coordination) and spatial memory in rats with middle cerebral artery occlusion-reperfusion were improved after progressive intensity aerobic training (Zhang et al., 2012).

On the basis of these findings, it appears that training might alternate between high- and low-intensity sessions or might progressively reach high-intensity to accentuate improvement of either cognitive or motor performance. It also suggested that treadmill training might be appropriate for improving these two functions. However, it is more difficult to find a compromise for the timing of training initiation. Indeed, an early training seems to be more appropriate to promote motor recovery while cognitive performances were improved when aerobic training started later. Therefore, the influence of aerobic training on cognitive deficits might be considered to complete the actual exercise recommendations.

## Methodological Considerations

Although it is currently admitted that aerobic training positively affects neuro-cognitive impairments, available studies reveal a great heterogeneity in the methods used and for some, weaknesses, which make results difficult to compare (Cumming et al., 2012). These methodological limitations, which are either specific or common to human and animal models, need to be considered before interpreting results.

### Methodological Considerations Concerning Exercise Parameters in both Animal and Human Studies

Available studies strongly differ in the parameters related to exercise during aerobic training: duration, intensity, frequency, mode and timing of rehabilitation initiation. In particular, exercise intensity, which is a critical parameter of aerobic training effectiveness, deserves to be questioned (Pin-Barre and Laurin, 2015). For both human and animal models, intensity is frequently based on empirical speed/power (Ploughman et al., 2008; Kluding et al., 2011; Påhlman et al., 2012; Shih et al., 2013; Shimada et al., 2013). In some human studies, the intensity was determined from subjective parameters such as level of exertion perceived by the patient (Ploughman et al., 2008; Kluding et al., 2011; Påhlman et al., 2012). In these conditions, exercise intensity (moderate, intense and severe) could not be precisely determined because no physiological markers were recorded (Xu and Rhodes, 1999). Therefore, a given absolute intensity was considered as moderate for some authors but as severe for others. When training intensity was based on physiological markers, percentages of maximal heart rate or the maximal oxygen uptake (VO_2peak_) were the most frequently used parameters (Ploughman et al., 2008; Quaney et al., 2009; Kluding et al., 2011; El-Tamawy et al., 2014). However, these methods are insufficiently reliable to distinguish between high and moderate training intensities. Indeed, patients barely reach their maximal aerobic capabilities during an incremental test. Recently, submaximal parameters such as ventilatory or lactic threshold have been recognized to be more suitable than VO_2peak_ to induce a higher inter-individual reproducibility in physiological response to exercise (Faude et al., 2009; Marzolini et al., 2013; Bosch et al., 2015). It is noticeable however that there is no consensus on the methods used to measure these physiological parameters from an incremental exercise test (Bentley et al., 2007). Indeed, depending on the type of incremental test, the performance and the related physiological parameters could be altered. The chosen ergometer (ergocycle or treadmill), stage duration as well as the magnitude of intensity increment between each stage are known to affect the performance (for review see, Bentley et al., 2007). Moreover, the progressive increase of exercise intensity on treadmill could be induced either by an elevation of speed (m/s) and/or inclination (percentage). The speed increment is not systematically reported in literature but some authors indicated that the grade increment was increased of 2% every 2 min with a constant (high) walking speed (Voss et al., 2013). Increase in the slope of the treadmill needs to be considered to improve the validity and the relevance of the chosen incremental test on treadmill for aging subjects. Indeed, the increment of treadmill inclination seems to be more appropriate for aging people and/or for the individuals for whom running is impossible or difficult. For instance, a lower running/walking speed on treadmill could reduce the perceived exertion of the exercise for some individuals and thus might reach highest intensities (Ehlen et al., 2011). Moreover, it was reported on obese persons that faster walking speeds might increase the risk of musculoskeletal injuries because of higher reaction forces and loading rates (Ehlen et al., 2011) in lower extremities tendons, joints and ligaments (Puga et al., 2012). Finally, most authors frequently used a stationary cycle ergometer for aging people (Maass et al., 2015) because measurements of physiological parameters during the test are more stable using this device compared to treadmill. The risk of falls is also lower on cycle ergometer. For the rodent model, some studies have proposed different treadmill protocol in order to reach the highest VO_2peak_ by modifying the treadmill inclination. It has been found in both rats and mice that the highest VO_2peak_ was reached at 25° because a distinct leveling-off of VO_2_ was mainly observed at this inclination (Wisløff et al., 2001; Kemi et al., 2002).

In addition, among different studies, intensities are rarely individualized, especially in rodents, while training individualization is one of the most important recommendations of stroke rehabilitation (Pang et al., 2013; Schmidt et al., 2014). This limitation might attenuate the “real” effectiveness of aerobic training. Finally, it is commonly considered in exercise physiology studies that energy expenditure needs to be equivalent between exercise types in order to compare the different effects of a specific training parameter (such as intensity or duration). In this way, all the experimental groups have the same energy expenditure and thus only the influence of a tested exercise parameter is assessed (Rognmo et al., 2004; Wisløff et al., 2007). However, it has never been applied in animal as well as in human stroke studies.

### Specific Methodological Considerations in Human

Inter-individual differences in cerebral ischemia location and/or aerobic fitness level may affect, positively or negatively, cognitive impairments. However, they remain difficult to counteract (Tang et al., 2013; Sun et al., 2014b). For example, it was found that patients with infarction located within cortical regions, middle cerebral artery territory and/or on left hemisphere were more prone to cognitive impairments (Sun et al., 2014b).

Otherwise, using a cognitive test that did not detect the specific cognitive impairments of a patient might hide some potential effect of a training intervention (McDonnell et al., 2011; Cumming et al., 2012). For instance, cognitive measurements are frequently limited to clinical tests, as functional independence measures (FIM), that are not enough sensitive. Likewise, mini-mental state examination (MMSE) may underestimate stroke-related cognitive deficits because it presents a lack of sensitivity for identifying disorders of visual perception and of high-order executive functions (Nys et al., 2006; Pendlebury et al., 2010; Cumming et al., 2012). This might be problematic given that these latter cognitive functions are frequently affected by stroke (Sun et al., 2014b; Tiozzo et al., 2015). In this respect, the MoCA can assess numerous cognitive impairments such as executive function, attention and delayed recall disorders that appear to be more suitable for stroke patient (Pendlebury et al., 2010). Some studies have also used specific neuropsychological tests such as Trail-making part A and B, Symbol digit test, Stroop test, Digit backward test, which allow to better detect cognitive deficits induced by stroke (Ploughman et al., 2008; Quaney et al., 2009; Rand et al., 2010; Kluding et al., 2011).

To ensure that cognitive performance improvements are related to aerobic training effectiveness, an increase of aerobic fitness needs to be observed at the end of the intervention. However, change in cardiorespiratory fitness after aerobic training is not systematically reported in the different studies. Thus, caution is often required when it is claimed that cognitive improvements are associated with aerobic training rather than other interventions or environmental factors.

The issue of cognitive-motor interactions in stroke patients also deserves to be considered. Indeed, cognitive and motor processes are classically considered as functionally independent and then, explored separately in the literature. However, the control and learning of complex goal-directed movements require a close cooperation between sensorimotor control processes and higher cognitive functions. This is even more marked in older adults, for which cognitive and motor processes become less differentiated by virtue of functional reorganization of brain activation patterns. Thus, change in cognitive-motor interplay expresses an important facet of age-related intrinsic plasticity of brain and cognition. Strategic variations might be thus analyzed to assess behavioral adaptability in cognitive (Lemaire and Hinault, 2014) and sensorimotor tasks (Poletti et al., 2015, 2016).

### Specific Methodological Considerations in Animal Models

Animal experiments can provide information about underlying mechanisms of neuroplasticity that could not be investigated in human. However, several drawbacks are often observed in some studies. For instance, it is impossible to investigate the large range of cognitive functions identified in human, such as verbal learning and memory for example (Voss et al., 2013). Cognitive dysfunctions after cerebral ischemia are limited to spatial and working memory, recognition and motor learning skills (Morris water maze, passive avoidance test, object recognition or location test; Luo et al., 2007; Griesbach et al., 2009; Cechetti et al., 2012; Shih et al., 2013; Shimada et al., 2013).

In addition, exercise-induced neuroplasticity are mainly explored within hippocampus (CA1 and CA3 areas and dentate gyrus), which is related to memory and learning (Vaynman et al., 2003). However, other areas such as basal ganglia, prefrontal cortex, thalamus and cerebellum are also involved in learning and memory processes, executive functions and motor control (Graybiel, 1995; Doya, 2000; Johnson and Ojemann, 2000). Except the hippocampus, other regions, remote away from the lesion zone, are connected to the affected structures and thus might also be disturbed after stroke (i.e., diaschisis effect and synaptic inhibition). For example, inflammatory responses could be observed within thalamus or substantia nigra after cortical brain injury that might partially contribute to explain the cognitive deficits (Block et al., 2005). It could be relevant to investigate the effect of aerobic training on the cognitive functions of these cerebral areas (Carmichael et al., 2004).

## Conclusion

This present article provides an overview of the positive effect of aerobic training on cognitive functions. It seems that training could increase the release of the same neurotrophic factors (BDNF and VEGF) in both elderly and stroke people that mediate beneficial neuroplasticity in brain areas involved in cognitive functions. We also identify some methodological limitations in both human and animal studies such as the standardization procedure of aerobic exercise intensity and the chosen cognitive tests, depending on the target population, that remain one of the most important concerns. Moreover, our review article suggests that the combination of aerobic training with other exercises/therapies or treatments represent a promising strategy with strong clinical perspectives. Importantly, this review highlights the lack of a firm guideline for exercise recommendations targeting recovery of cognition in stroke patient. Therefore, no standard aerobic protocol has yet been established as a commonly accepted reference regarding intensity, timing of training initiation and exercise type. It also appears that investigating changes in cognitive-motor interplay are critical to develop appropriate rehabilitation to improve both cognition and motor control after stroke.

## Author Contributions

All authors listed, have made substantial, direct, and intellectual contribution to the work, and approved its final version for publication. JL and J-JT: conceived the review focus, conducted literature review, summarized, and finalized the manuscript. PD: summarized, and finalized the manuscript. AC, CP-B and JL: reviewed literature, wrote first draft, and finalized the manuscript. All authors approved final version of manuscript.

## Funding

This work was supported by Aix-Marseille Université (AMU) and Centre National de la Recherche Scientifique (CNRS).

## Conflict of Interest Statement

The authors declare that the research was conducted in the absence of any commercial or financial relationships that could be construed as a potential conflict of interest.
